# Polyunsaturated fatty acids promote *Plasmodium falciparum* gametocytogenesis

**DOI:** 10.1242/bio.042259

**Published:** 2019-06-20

**Authors:** Takeshi Q. Tanaka, Suzumi M. Tokuoka, Daichi Nakatani, Fumie Hamano, Shin-ichiro Kawazu, Thomas E. Wellems, Kiyoshi Kita, Takao Shimizu, Fuyuki Tokumasu

**Affiliations:** 1 International Medical Zoology, Graduate School of Medicine, Kagawa University, Kagawa, 761-0793, Japan; 2 Laboratory of Malaria and Vector Research, National Institute of Allergy and Vector Research, National Institutes of Health, Bethesda, MD 20892-8132, USA; 3 Research Unit of Advanced Preventive Medicine, National Research Center for Protozoan Diseases, Obihiro University of Agriculture and Veterinary Medicine, Obihiro, Hokkaido 080-8555, Japan; 4 Department of Lipidomics, Graduate School of Medicine, The University of Tokyo, Tokyo, 103-0033, Japan; 5 Lipid Signaling Project, Research Institute National Center for Global Health and Medicine, Tokyo, 162-8655, Japan; 6 School of Tropical Medicine and Global Health, Nagasaki University, Nagasaki, 852-8523, Japan

**Keywords:** Malaria, Gametocyte, Phospholipids, Neutral lipids, Lipidomics, Mass spectrometry

## Abstract

The molecular triggers of sexual differentiation into gametocytes by blood stage *Plasmodium falciparum*, the most malignant human malaria parasites, are subject of much investigation for potential transmission-blocking strategies. The parasites are readily grown *in vitro* with culture media supplemented by the addition of human serum (10%) or by a commercially available substitute (0.5% AlbuMAX). We found better gametocytemia with serum than AlbuMAX, suggesting suboptimal concentrations of some components in the commercial product; consistent with this hypothesis, substantial concentration differences of multiple fatty acids were detected between serum- and AlbuMAX-supplemented media. Mass spectroscopy analysis distinguished the lipid profiles of gametocyte- and asexual stage-parasite membranes. Delivery of various combinations of unsaturated fatty-acid-containing phospholipids to AlbuMAX-supported gametocyte cultures improved gametocyte production to the levels achieved with human-serum-supplemented media. Maturing gametocytes readily incorporated externally supplied d5-labeled glycerol with fatty acids into unsaturated phospholipids. Phospholipids identified in this work thus may be taken up from extracellular sources or generated internally for important steps of gametocyte development. Further study of polyunsaturated fatty-acid metabolism and phospholipid profiles will improve understanding of gametocyte development and malaria parasite transmission.

## Abstract (precis)

**Summary:** Supplementation of extracellular lipids with unsaturated fatty acids can boost the production of *Plasmodium falciparum* gametocytes under laboratory culture conditions. Lipid uptake and metabolism are important processes for sustainable gametocytogenesis.

## INTRODUCTION

Malaria caused by *Plasmodium spp.* is a life-threatening infectious disease that killed 435,000 people in 2017, mostly young children infected by *Plasmodium falciparum* in Africa (World Health Organization, 2018). Artemisinin-combined therapies (ACTs) are currently the first-line antimalarial treatment worldwide, but the rising prevalence of ACT-resistant strains threatens the continuing effectiveness of these drugs (Saunders and Lon, 2016; Spring et al., 2015; World Health Organization Global Malaria Programme, 2017). Increased levels of gametocytemia have been reported with these strains (Ashley et al., 2014; Lozano et al., 2018), potentially compromising the ability of ACTs to reduce disease transmission by mosquitoes in programs of malaria control.

Interruption of gametocytogenesis with consequent elimination of transmission to mosquitoes is a potential strategy for malaria control (Goncalves et al., 2016; World Health Organization Global Malaria Programme, 2015). The process of gametocytogenesis begins with sexual-stage commitment of a blood-stage parasite (Filarsky et al., 2018; Josling et al., 2018). Ensuing gametocyte development times and the specifics of their morphology differ among the *Plasmodium spp.* (Alano, 2007; Baro et al., 2017; Lee et al., 2009). In the case of *P. falciparum*, trophozoite stage I forms are followed by stages II, III, IV and finally mature, circulating stage V gametocytes (Joice et al., 2014). The development period for *P. falciparum* gametocytes is about 10 days (Jeffery and Eyles, 1955).

*P. falciparum* gametocyte stages III–V are tolerant of many antimalarial compounds, however, novel gametocytocidal compounds have been recently identified by high-throughput screening systems (Sanders et al., 2014; Sun et al., 2017, 2014; Tanaka et al., 2013, 2015). Several potential drug targets are also reported (Sun et al., 2017, 2014), although much about the mechanisms of gametocytocidal activity is unclear. Indeed, understanding of cell biological and proteomic aspects of gametocyte stages remains limited, even though some genes and mechanisms involved in the transition from the asexual stages to the early stage I gametocytes have been identified (Brancucci et al., 2014; Chaubey et al., 2014; Coleman et al., 2014; Eksi et al., 2012; Filarsky et al., 2018; Ikadai et al., 2013; Josling et al., 2018; Kafsack et al., 2014). Unique features of energy metabolism and lipid biology have been reported in late-stage gametocytes (Lamour et al., 2014; MacRae et al., 2013; Tao et al., 2014; Tran et al., 2014). Factors involved in commitment and development of gametocytogenesis have also been described in mouse models with rodent-infective *Plasmodium* parasites (Boisson et al., 2011; Sinha et al., 2014; Waters et al., 1997). The morphology and developmental period of gametocytes in these models nevertheless differ from those of *P. falciparum*; large differences in these parameters also occur between *P. falciparum* and other human parasites such as *Plasmodium vivax*, despite the presence of evolutionarily-conserved features of gene transcription patterns in the early-, mid- and late-stage gametocytes of these species (Obaldia et al., 2018).

Growth and development of asexual intraerythrocytic stages depends upon the acquisition of extracellular lipids (Asahi, 2009; Asahi et al., 2011; Grellier et al., 1991; Mi-Ichi et al., 2006). AlbuMAX, a lipid-rich bovine serum albumin supplement, is often used to meet this requirement in lieu of human serum for cultivation of *P. falciparum in vitro*. Although AlbuMAX supplementation of the culture medium can maintain asexual-stage parasites in healthy states of *in vitro* propagation, its use has been associated with reduced presentation of PfEMP1 molecules on host erythrocyte membranes relative to the levels with human serum supplement, suggesting that externally supplied lipid components can influence host cell modification (Frankland et al., 2007). During gametocytogenesis, *P. falciparum* parasites undergo remarkable morphological changes and adopt elongated, falciform shapes. This structural transformation in the sexual stages is characterized by a multi-layer membrane complex with the parasite plasma membrane and accumulations of lipid bilayer material in mature gametocytes (Furuya et al., 2005; Sinden and Smalley, 1979). Fatty acid elongation enzyme and phosphoethanolamine methyltransferase are known to have critical roles in gametocyte development (Bobenchik et al., 2013; Ikadai et al., 2013), highlighting the importance of lipid metabolism in sexual stages. Late-stage gametocytes have also been shown to consume various lipid moieties from the culture medium (Lamour et al., 2014).

In this study, we were motivated by the differential effects of human serum or AlbuMAX to identify and examine key lipid components and their roles in gametocyte production. Unique lipid profiles of gametocytes and the contributions of extracellular factors to gametocytogenesis were previously reported (Brancucci et al., 2017; Gulati et al., 2015; Tran et al., 2016), raising questions about the the biochemical pathways and molecular developments involved. Here we show that gametocytes cultivated in the presence of human serum or AlbuMAX differ substantially in their content profiles of fatty acids in lipids including: diacylglycerols (DAG) and monoacylglycerols (MAG); phosphatidylcholine (PC) and phosphatidylethanolamine (PE) phospholipids; and polyunsaturated fatty acids (PUFA). Supplementations with certain PUFA can boost gametocyte production in AlbuMAX, suggesting possible targets in lipid pathways for further investigation.

## RESULTS

### Gametocyte production and fatty acid levels in human serum- and AlbuMAX-supplemented culture media

**Fig. 1.**
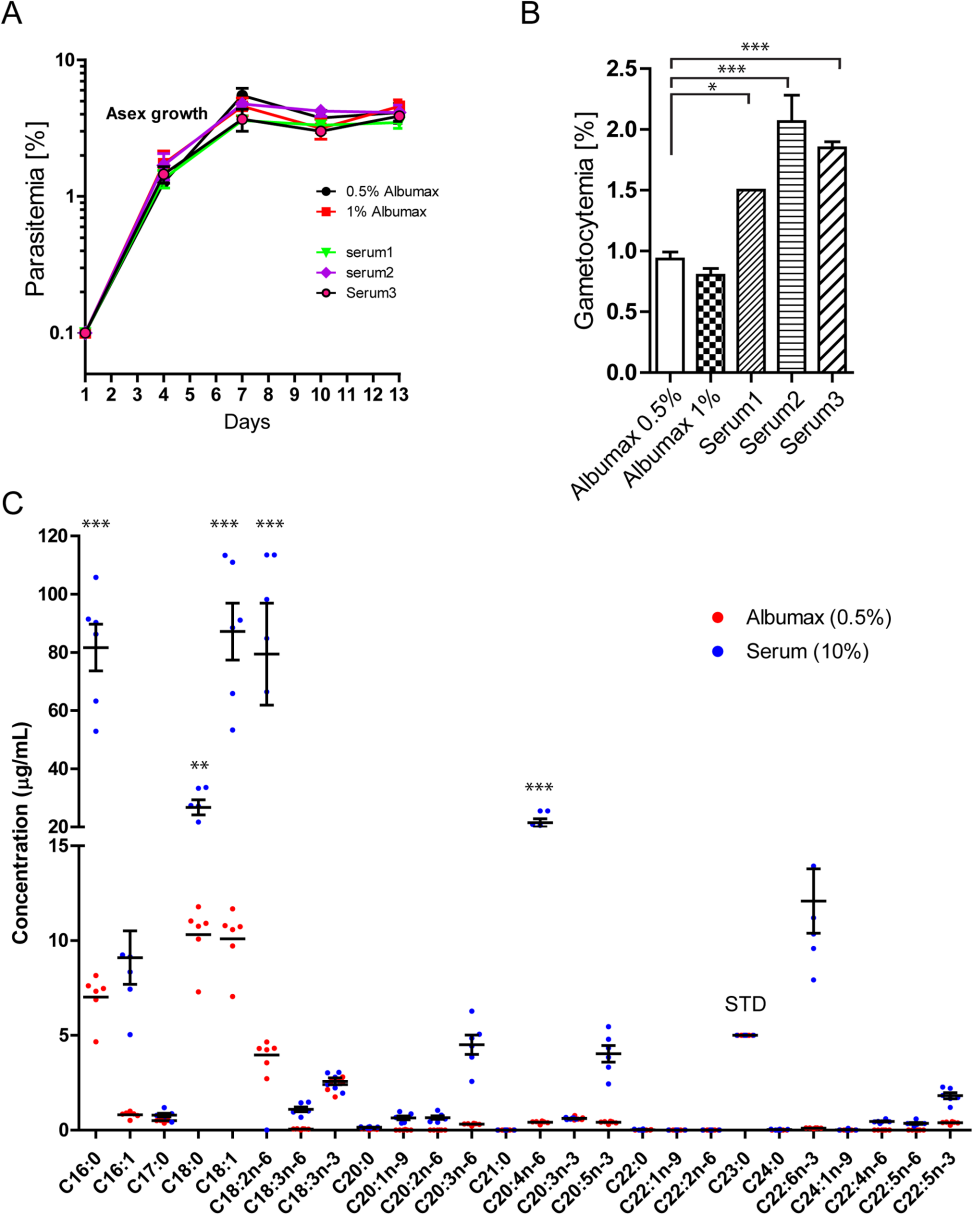
***P. falciparum* asexual-stage growth and gametocyte production in culture media with different lipid sources.** (A) Asexual growth with AlbuMAX or serum, monitored over 13 days. (B) Effect of lipid source on gametocyte production compared on day 16 with four independent parallel cultures (*n*=4, one-way ANOVA). (C) Fatty acid profiles of human serum and AlbuMAX analyzed by gas chromatography coupled to a flame ionization detector (GC-FID). Six independent batches of human serum and AlbuMAX were diluted in incomplete RPMI to the desired culture concentration (AlbuMAX: 0.5%, serum: 10%) in total 1 ml before sample processing. C23:0 (5 µg/ml) was added to the samples as an internal standard and other fatty acid concentrations were estimated based on the C23:0 signal. *P<0.05, **P<0.01, ***P<0.001 by two-way ANOVA with Bonferroni post-test, *n*=6. Asex, asexual stage parasites; STD, internal standard.

Tests of supplementation with three different sera and with 0.5% AlbuMAX showed indistinguishable curves of asexual-stage growth under standard culture conditions (Fig. 1A); however, induction of gametocytes was approximately 1.5–2.0× greater with all three sera than with the AlbuMAX (Fig. 1B). These results suggest a better representation in serum of factors advantageous to gametocytogenesis. We therefore used gas chromatography (GC) to compare incomplete RPMI media (1 ml) supplemented with standard conditions of 0.5% AlbuMAX or 10% human serum. Results (Fig. 1C) showed that the concentrations of many fatty acid species were lower in the AlbuMAX- than in the human serum-supplemented medium. For example, the common fatty acid C16:0 was present in AlbuMAX at less than one-tenth of the level in the human-serum-supplemented sample [7.01 µg/ml±0.50 µg/ml (AlbuMAX) versus 81.65 µg/ml±8.04 µg/ml (serum), mean±s.e.m., *n*=6]. Fatty acid C18:2 was present at ∼one-twentieth of the level in the human-serum-supplemented sample [4.00 µg/ml±0.29 µg/ml (AlbuMAX) versus 79.40 µg/ml±17.50 µg/ml (serum)]. Differences were even more remarkable with polyunsaturated fatty acids. Only very small amounts of long (over C20) polyunsaturated fatty acids were detected in culture medium supplemented with AlbuMAX (<1.00 µg/ml). For example, the levels of C20:4 and C22:6 were 0.41 µg/ml±0.03 µg/ml and 0.11 µg/ml±0.01 µg/ml, while serum-containing medium contained levels of 21.46 µg/ml±1.42 µg/ml and 12.09 µg/ml±1.70 µg/ml, respectively.

### Microscopic distributions of lipid and cholesterol indicators in asexual stages and gametocytes

**Fig. 2.**
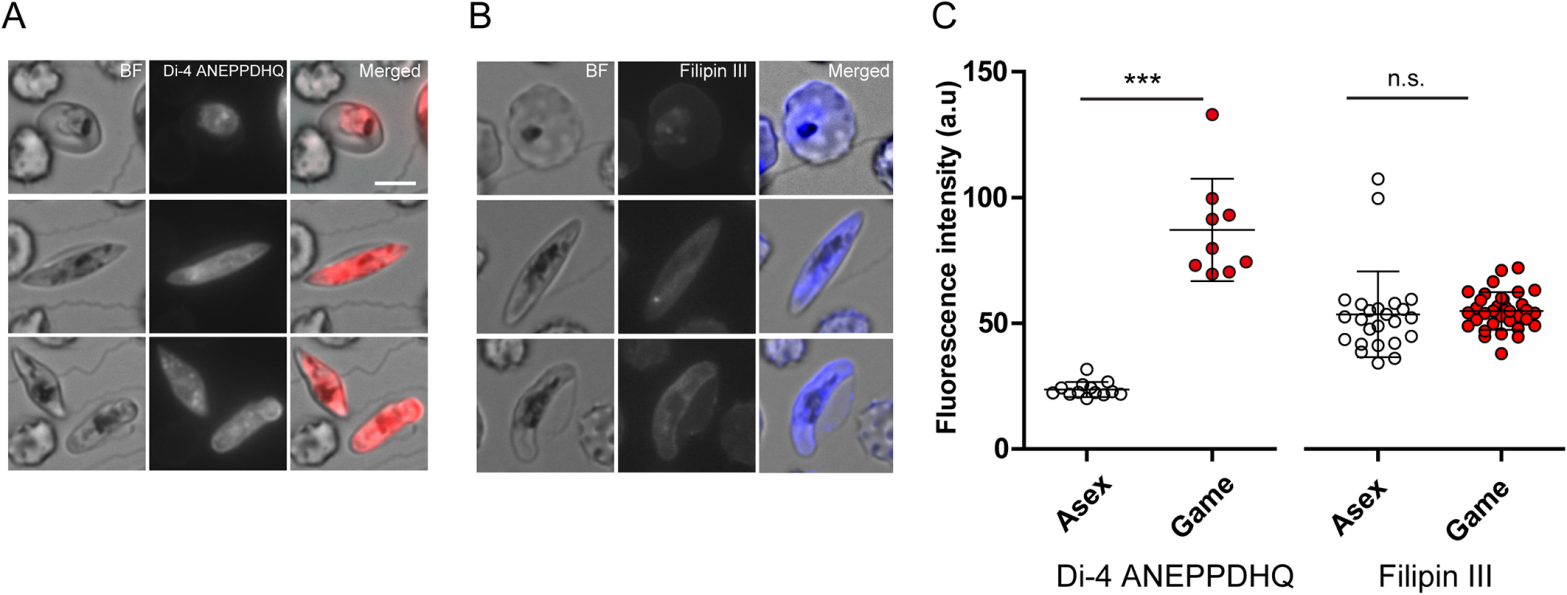
**Epifluorescence microscopy analyses of lipid distribution in parasitized erythrocytes.** (A,B) Lipophilic compartments (live cells) and cholesterol (fixed cells) detected by Di-4 ANEPPDHQ and Filipin III. (C) Averaged pixel intensity of fluorescence in each parasitized erythrocyte [*n*=13 (Di-4 asex), 9 (Di-4 gametocyte), 24 (Filipin III asex), 34 (Filipin III gametocyte)]. Bars indicate mean±s.d., and the differences in mean intensity were analyzed by unpaired *t*-test with Welch correction; ***P<0.001, n.s., not significant. Asex, asexual stage parasites; Game, gametocytes. Scale bar: 5 µm.

We next evaluated the microscopic distributions of lipids in asexual stages and gametocytes, using fluorescence indicators for lipids and cholesterol. The naphthylstyryl-pyridinium dye, Di-4 ANEPPDHQ, which serves as an indicator of both lipophilic compartments and cholesterol-rich membranes (Obaid et al., 2004; Tokumasu et al., 2014), showed greater epifluorescence from gametocytes than from trophozoites grown with human serum supplement (Fig. 2A). Such a difference of intensity was not observed with Filipin III, which is specific for the detection of cholesterol (Fig. 2B). Quantitation in terms of average signal strength per pixel indicated an estimated 3.6-fold lower signal from Di-4 ANEPPDHQ in the asexual stage trophozoites than in gametocytes, whereas no significant difference in signal was found with Filipin III (Fig. 2C). Taken together, these results suggest a higher fraction of non-cholesterol lipids relative to cholesterol content in the gametocytes.

### Principal component analysis of non-cholesterol lipids in asexual stages and gametocytes

**Fig. 3.**
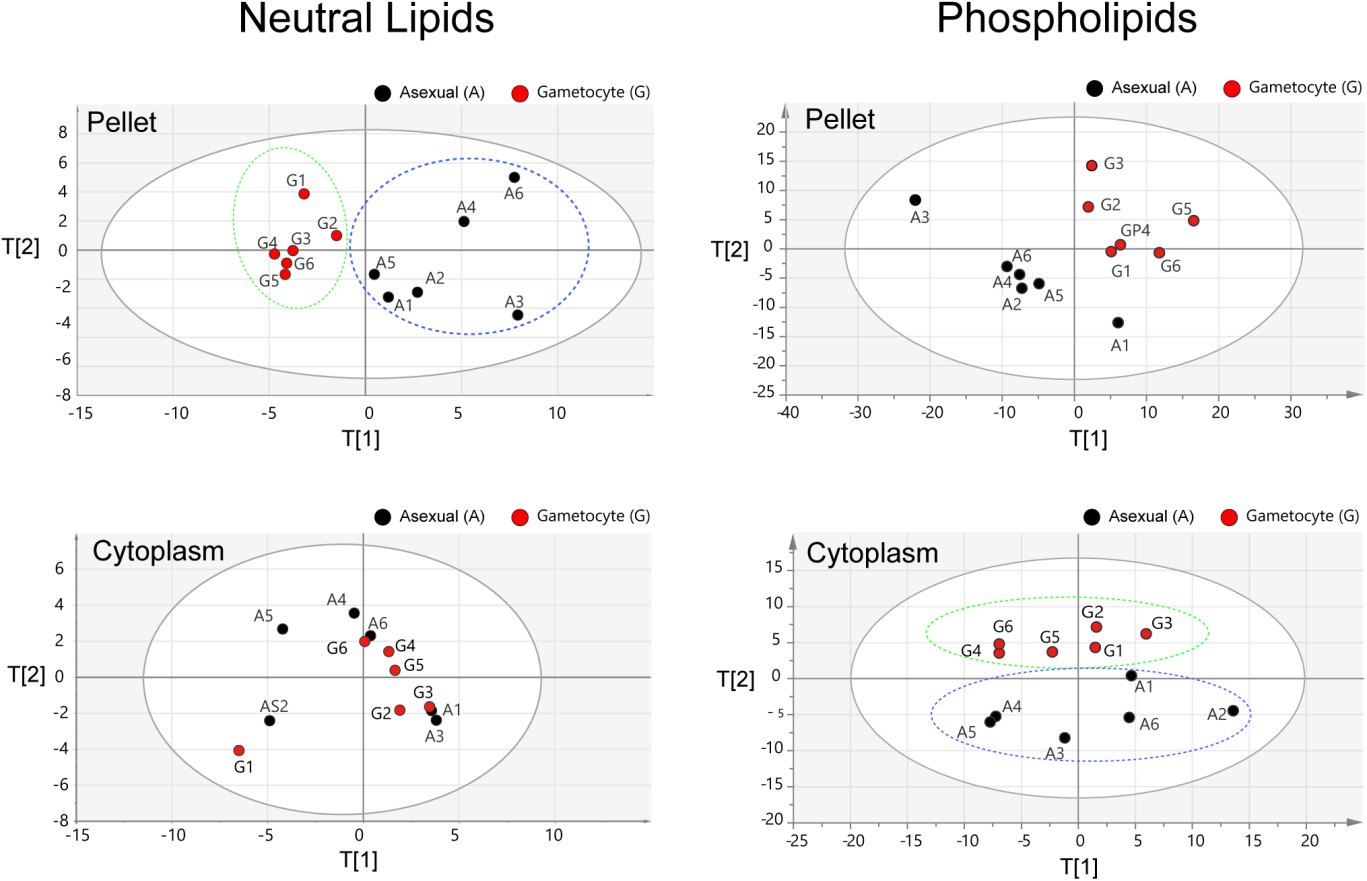
**PCA analyses (score plot for the first two components) of neutral and phospholipid profiles of six independent data sets (**Figs 4 and 5). T[1]: first component; T[2]: second component. Fractional data sets from asexual and gametocyte stages were analyzed by SIMCA software. The numbers represent the sample IDs.

To obtain an overall comparison of non-cholesterol lipids in gametocytes versus asexual parasites, and preliminary analyses of individual lipid species, we applied principal component analysis (PCA) to overall LC-MS data from the different parasite stages. These data were collected from the cell membrane pellet and cytoplasm samples of six independent cultures supplemented with different sera. Fractional values were calculated for 61 mono- or diacylglycerides (MAG, DAG; neutral lipids) and for 347 species for phospholipids in each the datasets

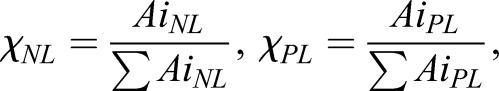

where: *χ* is the fraction of lipid; *NL* is the fraction of neutral lipid; *PL* is the fraction of phospholipid and *Ai* is the area value of mass chromatogram for each lipid). Analysis of triacylglycerides (TAG) was not included because of unavailability of reference TAG samples covering a large variety of fatty acid combinations. In PCA of the pellet samples, neutral lipids showed a clear separation between the data points of asexual and gametocyte stages along the first principal axis, whereas the phospholipids of the different stages grouped into two different areas of the plane of principal axes 1 and 2 (Fig. 3, upper panels). In contrast, PCA of the cytoplasmic samples showed no clear separation between the neutral lipid data points of asexual stages and gametocytes; however, separation was evident between the phospholipid datapoints of these stages along principal axis 2 (Fig. 3, lower panels). These results suggest characteristic profiles of the lipid types in asexual stages and gametocytes, consistent with distinct compositions of the membranous and intracellular compartments of these stages.

### Neutral lipid profiles of asexual stages and gametocytes

**Fig. 4.**
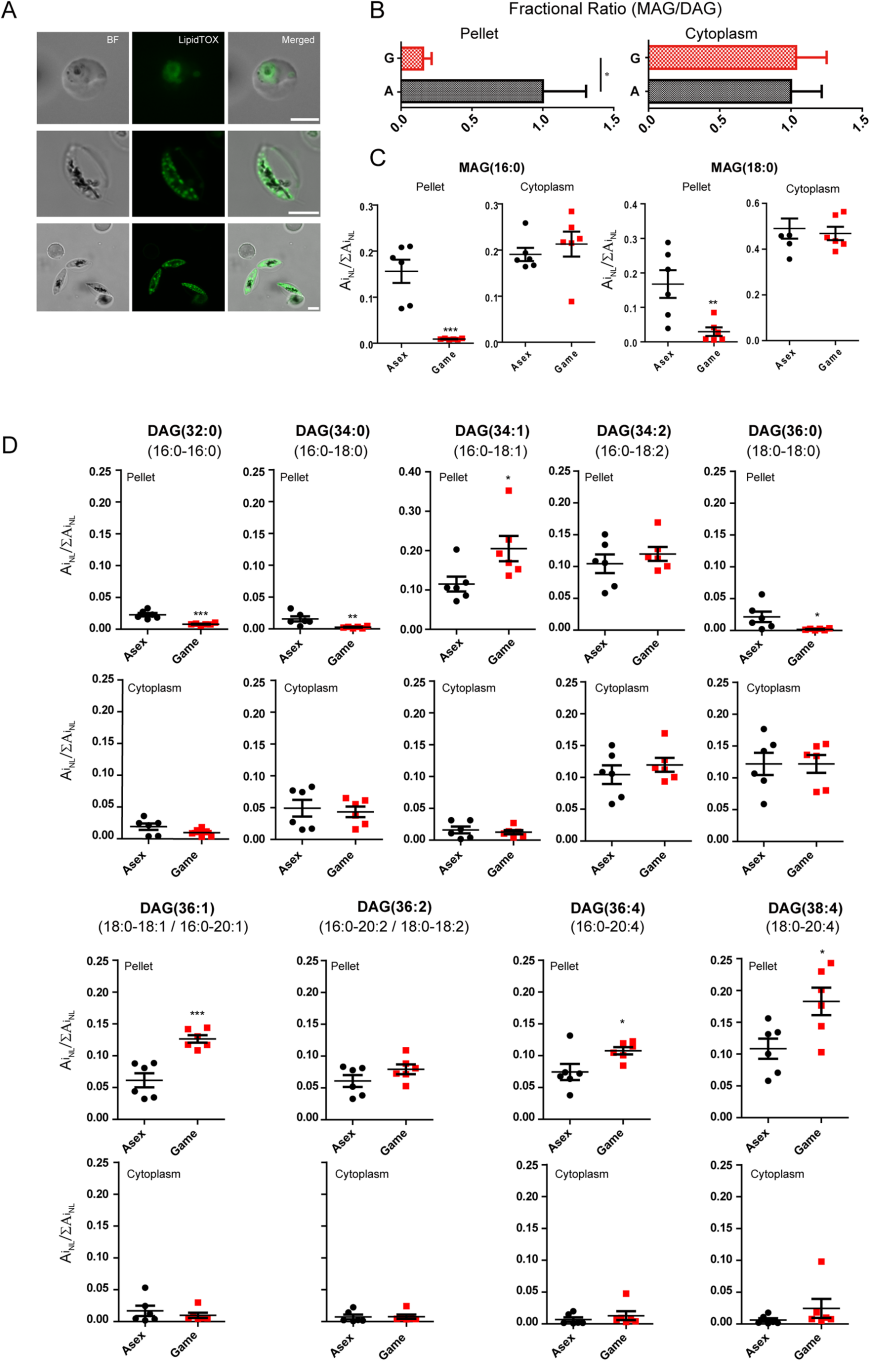
**Neutral lipid profiles of asexual and gametocyte stages.** (A) Confocal fluorescence microscopy analysis of neutral lipid distribution probed by LipidTOX neutral lipid indicator. (B) The sum of MAG fraction (against total neutral lipid) was normalized against the total DAG (*n*=6). (C,D) Stage-dependent changes in individual MAG and DAG species detected by LC-MS analysis on six independent cultures. Data are shown as fractions of each lipid against total neutral lipid and expressed as mean±s.e.m. (*n*=6) *P<0.05, **P<0.01, ***P<0.001 by two-tailed *t*-test. Scale bars: 5 µm.

To further study the differences in non-cholesterol lipids between asexual stage trophozoites and gametocytes, we evaluated fluorescence signals from LIPIDTOX, a marker of neutral lipids in cells. Patterns from these signals (Fig. 4A) showed droplet-like accumulations predominately near the periphery of the gametocytes under the parasite plasma membrane. Such accumulations were not detected in trophozoites, which instead showed a more uniform signal from the parasite cytoplasm with a few areas of accumulation in the host erythrocyte. Similar patterns of neutral lipid accumulation in gametocytes were reported from another study (Tran et al., 2016). We next used LC-MS to evaluate MAG and DAG in the cytoplasmic and pelleted fraction of gametocyte and trophozoite preparations. In this analysis, the ratios of fractional area values, MAG/DAG (sum of MAG/sum of DAG), were indistinguishable between the cytoplasmic preparations, whereas the MAG/DAG ratio was smaller in the pelleted fraction of gametocytes relative to trophozoites (Fig. 4B). In view of the higher levels of non-cholesterol lipids and LIPIDTOX-positive accumulation in gametocytes, these findings indicate an overall greater presence of DAG in the pelleted fraction from gametocytes. In contrast to the findings for DAG, two species of MAG, 16:0 MAG (palmitoyl glycerol) and 18:0 MAG (stearoyl glycerol), were found to be markedly higher in the pellets of asexual stages versus those of gametocytes (*P*=0.0141 and *P*<0.01, respectively) (Fig. 4C, Table S1).

Fig. 4D presents comparative levels of nine DAG species in the cytoplasmic and pelleted fractions from asexual and gametocytes. Little or no difference was evident in the cytoplasmic levels of almost all of these species, consistent with the similar MAG/DAG ratios of asexual stage and gametocyte cytoplasm (Fig. 4B). In the pelleted fractions, the level of saturated 32:0 (*P*=0.0002, *n*=6), 34:0 (*P*=0.0088, *n*=6) and 36:0 DAG (*P*=0.0388, *n*=6) was somewhat higher in asexual stages than gametocytes, whereas the levels of unsaturated 34:1 (*P*=0.0362, *n*=6), 36:1 (*P*=0.0004, *n*=6), 36:4 (*P*=0.0370, *n*=6) and 38:4 DAG (*P*=0.0197, *n*=6) was greater from gametocytes (Fig. 4D, Table S1)). Low MAGs (Fig. 4C) along with these higher DAGs account for overall low MAG/DAG ratio from gametocyte pellets (Fig. 4B). The differences in normalized fractional values were also evident in some phospholipid species (Fig. S1, Table S2 and S3). Our data show that lipid profile changes between asexual and gametocyte stages occur with phospholipids as well as neutral lipids, and that the fractional values of these changes depend on the acyl chain length and saturation level, as demonstrated previously (Tran et al., 2016).

### Externally-supplied polyunsaturated fatty acids can support gametocytogenesis

**Fig. 5.**
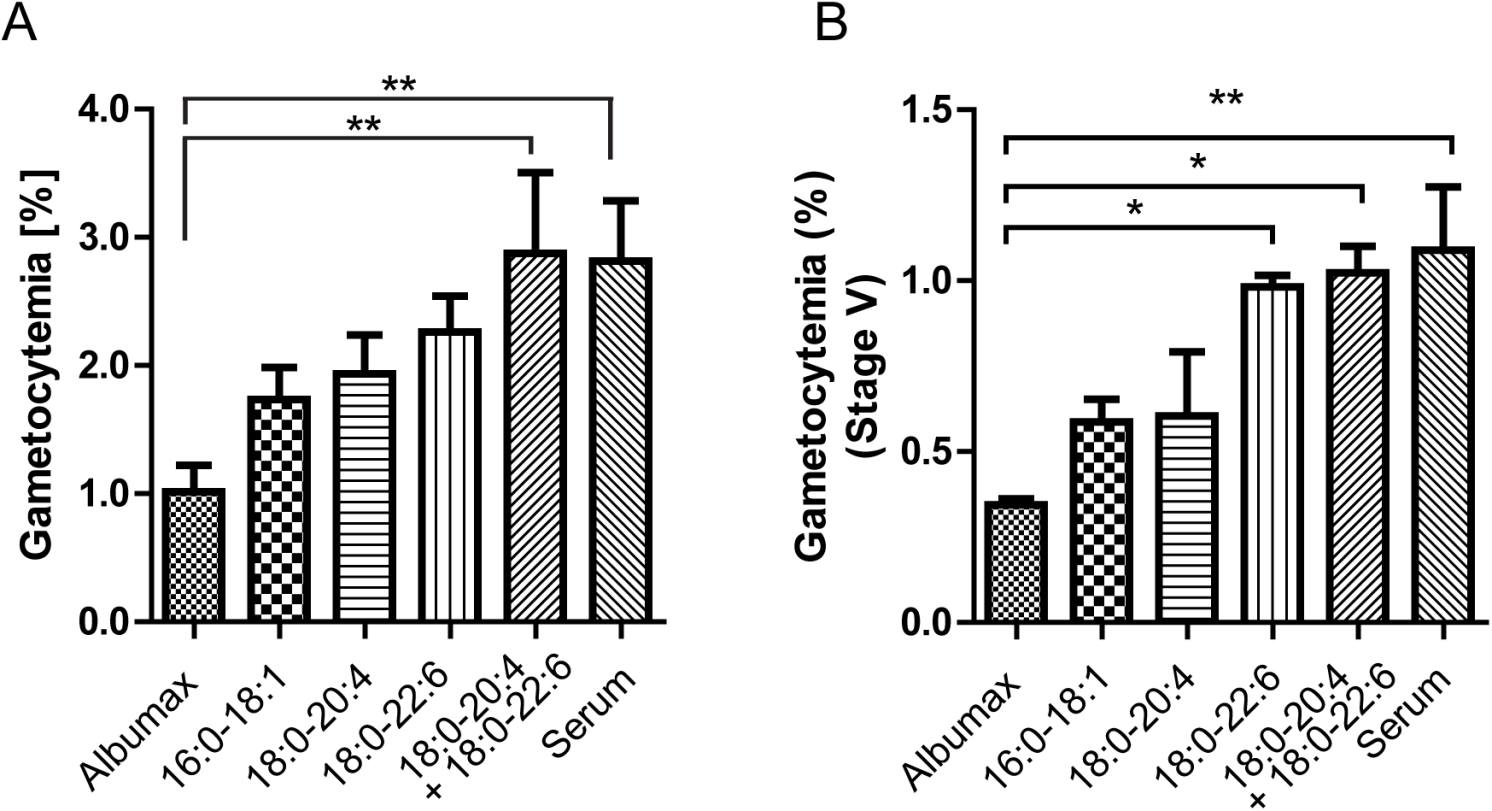
**Effects of PUFA-containing phospholipids in AlbuMAX-supported gametocyte culture (*n*=3 independent duplicated culture sets).** SUV with equal amounts of PC and PE containing the same fatty acids shown were added daily to total 1 ml of gametocyte cultures from day 1 until sample collection. (A) Total and (B) stage V gametocytemia were obtained as averaged values from duplicated samples in each parallel culture set and used for statistical analysis (one-way ANOVA with Dunnett's correction, *n*=3); *P<0.05, **P<0.01.

The above results suggested that PUFA addition might improve *P. falciparum* gametocytogenesis in AlbuMAX-supplemented culture medium. To evaluate this hypothesis, we performed experiments with various fatty acids that are abundant in human serum, including such saturated and unsaturated fatty acids as C16:0, C18:0, C18:1, C20:4 and C22:6. We first added fatty acids as stocks in ethanol/chloroform directly to the AlbuMAX-supplemented culture medium, to bring C20:4 and C22:6 to the same level as in human serum, but attempts to induce gametocytogenesis with this exposure for 13 days were unsuccessful (Fig. S2). Parallel asexual cultures showed a decrease in parasitemia under such conditions. We suspected that these were negative effects resulting from either the bare nature of supplied lipid or the organic solvent. To avert these effects, we arranged to instead supply the phospholipids in vesicle form. Small unilamellar vesicles (SUV) with equal amounts of PC and PE as 16:0–18:1 (POPC and POPE), 18:0–20:4 (SAPC and SAPE), 18:0–22:6 (SDPC and SDPE) or both 18:0–20:4 and 18:0–22:6 were prepared in incomplete RPMI plus 0.5% AlbuMAX and added every day from the start (day 0) of gametocyte induction. The added amounts approximately complemented one-half of the differences of fatty acid content between the 10% human serum and 0.5% AlbuMAX supplementation: ∼25 µg/ml for 16:0, ∼10 µg/ml for 18:0 and 20:4 and ∼5 µg/ml for 22:6 (Fig. 1C), representing moderate amounts to avoid over-supplementation that could cause adverse effects on cell membranes. Results of these experiments showed that SUV containing equal amounts of PC and PE in both their 18:0–20:4 and 18:0–22:6 forms were able to support increases in gametocytemia to the ∼3%, similar to those obtained with the human serum-supplemented conditions (Fig. 5A). Increases to 1.5–2.0% levels were obtained with SUV 16:0–18:1 (supplied in the highest amount to the culture), and to ∼2% levels with SUV 18:0–20:4 or 18:0–22:6. For stage V gametocytes, the 18:0–20:4 and 18:0–22:6 mixture boosted levels to those obtained with human serum (Fig. 5B); SUV of PC and PE supplied as only 18:0–22:6 forms raised stage V production to nearly the same range. Lower levels of stage V gametocytemia improvement were obtained with the 18:0–20:4 and 16:0–18:1 SUV (Fig. 5B). Addition of C22:6 may thus have been particularly effective for the realization of stage V forms.

## DISCUSSION

Results of this study show that certain PUFA can boost gametocyte production in 0.5% AlbuMAX-supplemented RPMI medium to levels that are obtained with 10% human serum supplement. Previous reports have characterized various lipid species in the development of gametocyte stages or in progress through the course of gametocytogenesis (Brancucci et al., 2017; Gulati et al., 2015; Tran et al., 2014, 2016). Brancucci et al. (2017) reported that media depleted of lysophosphatidylcholine may have been associated with a stimulation of gametocytogenesis. Our data now indicate that certain unsaturated fatty acid-containing phospholipids can promote gametocytogenesis, suggesting that gametocytogenesis is a complex phenotype under the influences of multiple lipids and perhaps other molecular species in the media environment. These observations also highlight the importance of extracellular lipids to the sexual development of *P. falciparum*. Increased signal from fluorescence lipid analogue ANEPPDHQ and the evidence for neutral lipid-rich accumulations at the gametocyte periphery affirm the build-up of lipids in gametocytogenesis. Enrichments of neutral lipids, such as DAG34:1 and DAG36:1, are consistent with the findings of Tran et al. (2016); our observation of these increases in the pellet, but not the cytoplasmic fraction, suggests that accumulations of these molecules are bound to membranes or localize to insoluble compartments. The mechanism and purpose of such accumulations of neutral lipid-rich concentrates remain to be established.

Differences are present between the phospholipid fractions of gametocytes and asexual-stage parasites. Phospholipid composition can influence cell development and survival by its effect on nanodomain formations as well as biophysical properties of membrane viscoelasticity and molecular diffusion (Epand, 2007; Pathak and London, 2015; Ramadurai et al., 2010; Vanni et al., 2014). Among malaria parasites, optimum cell membrane properties and their corresponding lipid profiles may differ between non-dividing gametocytes and the asexual-stage parasites that replicate in the blood stream. Since lipid species with saturated fatty acids can contribute to membrane rigidity, the relatively lower levels of MAG in gametocytes may be a factor in their flexibility to negotiate the filtration action of the spleen as they circulate in the bloodstream. Less membrane flexibility of mature asexual schizonts, which avoid splenic clearance by cytoadhering/sequestering in deep tissue microvasculature beds, may have features in common with the increased stiffness of HeLa cells that accumulate saturated phosphatidic acids, phosphatidylinositols, and ceramides prior to division (Atilla-Gokcumen et al., 2014).

Our molecular labeling data demonstrate different fatty acid incorporation patterns by the asexual stages and gametocytes: within a 24 h period, relative fractions d5-labeled lyso- and diacylphospholipids were significantly higher in the gametocytes for both PC and PE, with PE incorporation somewhat more pronounced (Fig. S3). In addition, many d5-labeled diacylphospholipids are unsaturated. A possible explanation for these observations is that *P. falciparum* phospholipid synthesis may be uniquely tuned to meet specialized fatty acid requirements of non-dividing gametocytes. Phospholipid metabolic pathways of apicomplexan parasites exhibit differences from those of humans as well as other parasites such as kinetoplastida (Ramakrishnan et al., 2013). PC in *P. falciparum* is synthesized through a CDP-choline and CDP-ethanolamine pathway (Kennedy pathway) including a conversion of ethanolamine-phosphate to choline-phosphate by *P. falciparum* phosphoethanolamine N-methyltransferase (PfPMT) (Pessi et al., 2005, 2004). Concentration-dependent inhibition of this conversion by the product choline-phosphate may therefore help to control the balance of these two molecules (Pessi et al., 2004).

## CONCLUSION

Extracellular lipids including particular PUFAs promote gametocytogenesis in AlbuMAX-supplemented culture medium. Accumulation of lipid material in gametocytes and the effects of externally supplied fatty acids on the parasite lipid profiles also suggest an adaptive preparation for mosquito infection, where the environmental medium and nutrition are greatly different from the mammalian bloodstream. Further understanding of the effects of extracellular lipid compositions and PUFA profiles may improve studies of gametocyte development and transmission.

## MATERIALS AND METHODS

### Parasite culture and synchronization

Erythrocytes and human sera were purchased from Interstate Blood Bank (Memphis, TN, USA), Japanese Red Cross Kanto-Koshinetsu Block Blood Center, Tokyo, Japan (No: 28J0058), and Hokkaido Block Blood Center, Sapporo, Japan (No: 25J0074). Asexual stages of *P. falciparum* parasite lines NF54 and 3D7 (a clone of NF54) (Walliker et al., 1987) were standardly cultivated in incomplete RPMI [consisting of RPMI 1640 (Invitrogen) plus 25 mM HEPES, 0.8 mg/ml L-glutamine, 0.05 mg/ml hypoxanthine (Sigma-Aldrich), 2 mg/ml sodium bicarbonate (Invitrogen), and 10 µg/ml gentamicin (Invitrogen)], supplemented with 10% O- or A-positive human serum as originally described (Trager and Jensen, 1976). Cultures were maintained at 37°C under an atmosphere of 5% O_2_, 5% CO_2_ and 90% N_2_. Thin films of erythrocyte samples were prepared, fixed with methanol, stained with Giemsa, and microscopically evaluated by standard methods (Moll et al., 2013). For preparation of trophozoite-rich culture, the asexual parasites were synchronized twice by sorbitol treatment (50 mg/ml D-sorbitol, Sigma-Aldrich) at a 7–10 h interval. The trophozoite-rich parasites were collected at 14–16.5 h after the second synchronization. Malaria parasite culture using human erythrocytes was carried out under approvals of ethical review by ethics committees of University of Tokyo (#10050 and #11064) and Obihiro University (#2013-04-2).

### Preparation of cytoplasmic fraction from parasites

Trophozoite-rich asexual parasites or late-stage gametocytes (day 15–16) were pelleted by centrifugation and washed three times with phosphate buffered saline (PBS), (140 mM NaCl, 2.7 mM KCl, 10 mM PO_4_^3−^, pH 7.4) (Takara Bio Inc., Kusatsu, Japan). Host erythrocyte membranes were obtained by lysis at 37°C for 10 min with 0.075% saponin/PBS solution, followed by centrifugation at 10,000×***g*** for 30 s. After removal of supernatant, the pellet was washed with three times PBS and once with HEPES buffer (10 mM HEPES, 140 mM NaCl, 10 mM glucose; pH 7.4). Lysis of the freed parasites was achieved by addition to the pellet of 30 pellet volumes of lysis buffer (10 mM Tris-HCl, 1 mM EDTA; pH 8.0) and vortexing. After centrifugation at 14,000 rpm for 15 min, cytoplasmic and membrane fractions were collected as the supernatant and pellet fractions, respectively.

### Gametocyte inductions and preparations

Gametocyte inductions (Tanaka and Williamson, 2011) were set up at 0.1% parasitemia, hematocrit (Hct) 6% on day 1 and maintained at Hct 3% from day 3 onwards. Late-stage gametocytes were enriched by 3 days of treatment (days 9–11) with 50 mM N-acetylglucosamine (NAG) to eliminate asexual stages, then purified by 65% Percoll density gradient centrifugation on day 12 (Tanaka and Williamson, 2011). The purified gametocytes were maintained by daily media change until the further analyses.

For assays of induced gametocytes, parental non-synchronized asexual parasites were plated in duplicate into 12 or 24-well plates at 0.1% parasitemia (Hct 6%) in incomplete RPMI supplemented with 0.5% AlbuMAX, 10% human serum, or phospholipid (PC, PE) concentrations indicated in the text. Phospholipids were purchased from Avanti Polar Lipids, Inc. (Alabaster, AL, USA).

### Vesicle preparations

Small unilamellar vesicles (SUV) were prepared by a conventional sonication method (Huang, 1969) with slight modification. In brief, 3 mg (16:0-18:1, PC and PE) or 1 mg (18:0-20:4 and 18:0-22:6, PC and PE) of lipids in ethanol/methanol (1:1) were dried in an EC-95C3T centrifuge concentrator equipped with cold trap (Sakuma, Tokyo, Japan), and re-hydrated with 1 ml of the incomplete RPMI with 0.5% AlbuMAX. After vortexing and sonication in an ultrasonic water bath, dispersed lipids were further sonicated by Tomy-Seiko UR-20P probe sonicator (Tomy-Seiko, Tokyo, Japan) at maximum setting and stored at 4°C. The differences in each fatty acid amount between human serum and AlbuMAX, i.e. 50 µg/ml for C16:0, 20 µg/ml for C18:0 and C20:4 (arachidonic acid), and 10 µg/ml for C22:6 (docosahexaenoic acid), were supplemented with an equal portion of PC and PE. Daily media changes were started on day 3, and asexual parasitemias and gametocytemias were determined from microscopic counts of Giemsa-stained smears.

### Fluorescence microscopy analyses

Parasitized erythrocyte samples at ∼0.1% Hct were immobilized onto a cleaned cover glass (No. 1.5) coated with 0.1 mg/ml type-I collagen (Chrono-Log, Havertown, PA, USA) (Calvo et al., 2010; Tokumasu et al., 2005). Loosely attached erythrocytes were gently washed with warm incomplete RPMI and crosslinked for 1 h with 50 mM dimethylsuberimidate (Sigma-Aldrich) in 0.1M sodium borate buffer, pH 9.5, containing 1 mM MgCl_2_. The reaction was immediately quenched by immersing the cells into 0.1M glycine in PBS, pH 7.4 for 1 h, to saturate unreacted aldehyde groups that produce background fluorescence. Live erythrocytes were stained for 30 min with 2 µg/ml Di-4 ANEPPDHQ (Invitrogen) at 0.1% Hct (Tokumasu et al., 2014) and washed with PBS. To detect cholesterol, Filipin III (0.4 mg/ml in DMSO/PBS) was applied and incubated for 1 h to fixed erythrocytes immobilized on the cover glass, followed by rinsing with PBS. For neutral lipid observations, parasitized erythrocytes at 0.1% Hct were stained with LipidTOX^TM^ (1:100) (Invitrogen) in incomplete RPMI for 30 min at 37°C; from this suspension, 4 µl were applied onto a clean slide glass and sealed by a coverslip with a high-vacuum grease (Corning, NY, USA) at four edges.

Di-4 ANEPPDHQ and Filipin III stained cells were imaged with Leica DMI6000B inverted fluorescence microscope (Leica Microsystems, Bannockburn, IL, USA) using 100× objective (N.A. 1.30) and custom-ordered filter cube with XF1073 (excitation 475AF40) and XF3081 (emission 645AF75) filters for Di-4, and an XF02 (excitation/emission 330WB80/400ALP) filter for Filipin III (Omega Optical, Brattleboro, VT, USA). Images (1344×1024 pixels, 8-bit grey scale) were captured with ORCA-ER digital camera (Hamamatsu Photonic Systems, Bridgewater, NJ, USA) controlled by ImagePro 5.1 software (Media Cybernetics, Silver Spring, MD, USA). LipidTOX signals were detected with a Leica SP5 confocal microscope with 63× objective lens (N.A. 1.32); excitation was with an Ar laser line at scan speed of 400 Hz. Emission was measured over a bandwidth of 511–555 nm, and frame/line averaging was set as 4. Captured images (1024×1024 pixels, 8-bit per channel) were exported as TIFF files by LAS X (Leica Microsystem) and analyzed by ImagePro 6.3 (Media Cybernetics) and GraphPad Prism 5 software (La Jolla, CA, USA).

### High performance liquid chromatography-mass spectroscopy and principal component analysis

Phospholipids and neutral lipids were extracted by MeOH and acetonitrile (Fujifilm-WAKO Pure Chemical, Osaka, Japan), respectively, with 10× sample volume. After incubation for 10 min at room temperature, the extracts were centrifuged at 4°C for 10 min at 18,700×***g***, and supernatants were collected and further diluted in MeOH or acetonitrile before liquid chromatography- electrospray ionization-mass spectroscopy (LC-MS) analysis. Neutral lipid and phospholipid LC-MS was performed using a Nexera Ultra High Performance Liquid Chromatograph (UHPLC) system and a triple quadrupole mass spectrometer LCMS-8050 or LCMS-8040 (Shimadzu Corp., Kyoto, Japan). Acquity UPLC BEH C8 (1.7 µm, 2.1 mm×100 mm) (Waters, Milford, MA, USA) column was used for reversed phase liquid chromatography with three phases: 5 mM NH_4_HCO_3_/water (mobile phase A), acetonitrile (mobile phase B), and isopropanol (mobile phase C). The pump gradient [time (%A/%B/%C)] was programmed as follows: 0 min (75/20/5)-20 min (20/75/5)-40 min (20/5/75)-45 min (5/5/90)-50 min (5/5/90)-55 min (75/20/5). Injection volume was 5 μl. Column oven temperature was set at 47°C and the flow rate was 0.35 ml/min.

SRM (selection reaction monitoring) transitions for phospholipids were set as follows: PC ([M+H]→184.1, positive mode), PE ([M+H]→[M+H]–141, positive mode), PS ([M-H]→[M-H]– 87, negative mode), PI ([M-H]→241, negative mode), where M are *m/z* of molecular related ions and acyl chains of carbon 12 to 24 in length were targeted. Sum of all area of chromatograms from these SRM transitions were used as ‘total area value for phospholipid’ to calculate fractional values. For MAG, acyl chains of 16 to 24 carbons in length were targeted and SRM transitions in negative ion mode set as [M-H]→FA. For DAG, acyl chains 16 to 22 carbons in length were targeted and SRM transitions in positive ion mode set as [M+NH_4_]→[(M+NH_4_)-NH_3_-FA]. Sum of all area of chromatograms from these SRM transitions were used as ‘total area value for neutral lipid’ to calculate fraction. PCA (Principal Component Analysis) was performed by SIMCA ver. 13.0 (Umetrics, Umeå, Sweden). Original data were scaled to unit variance in the analysis.

### Gas chromatography

For total fatty acid analysis of culture media, 1 ml of incomplete RPMI media containing 10% serum or 0.5% AlbuMAX was freeze dried, and C23:0 fatty acid (Sigma-Aldrich) was added to the dried samples as an internal standard. The samples with the internal standard were derivatized by the Fatty Acid Methylation Kit and purified by the Fatty Acid Methyl Ester Purification Kit (both from Nacalai Tesque, Inc., Kyoto, Japan). The fatty acid methyl ester samples were concentrated by evaporation at reduced pressure and dissolved with 25 μl of dichloromethane for GC-FID (Gas chromatography coupled to a Flame Ionization Detector) analyses by GC2010 Ultra (Shimadzu) equipped with FAMEWAX capillary column (30 m×0.25 mm I.D. ×0.25 μm d.f., RESTEK, Bellefonte, PA, USA). The injection port temperature was set at 240°C and a 2 ml aliquot was injected in the split mode (1:25). The column temperature was controlled as follows: the starting temperature was 140°C, and increased at 11°C/min to 200°C, 3°C/min to 225°C, and 20°C/min to 240°C, and finally maintained at 240°C for 5 min. Helium was used as a carrier gas with a constant linear velocity of 45 cm/s. Identification of fatty acid methyl ester was based on the retention time by comparing with a standard mixture (Supelco 37-FAME Mix, Sigma-Aldrich), C22:5 n-6 methyl ester (Nu-Chek Prep, Inc., Elysian, MN, USA), C22:5 n-3 methyl ester (Sigma-Aldrich) and C22:4 n-6 methyl ester (Cayman, Ann Arbor, MI, USA). The amounts of 26 fatty acid species and C23:0 internal standard were estimated by the standard curve and normalized against C23:0.

## Supplementary Material

Supplementary information
