# Supplementary material for: Polyunsaturated fatty acids promote Plasmodium falciparum gametocytogenesis
Source: Biol Open. 2019 Jun 20;8(7):bio042259. doi: 10.1242/bio.042259 (PMC6679406; doi:10.1242/bio.042259)
Supplement: Supplementary information [file biolopen-8-042259-s1.pdf]

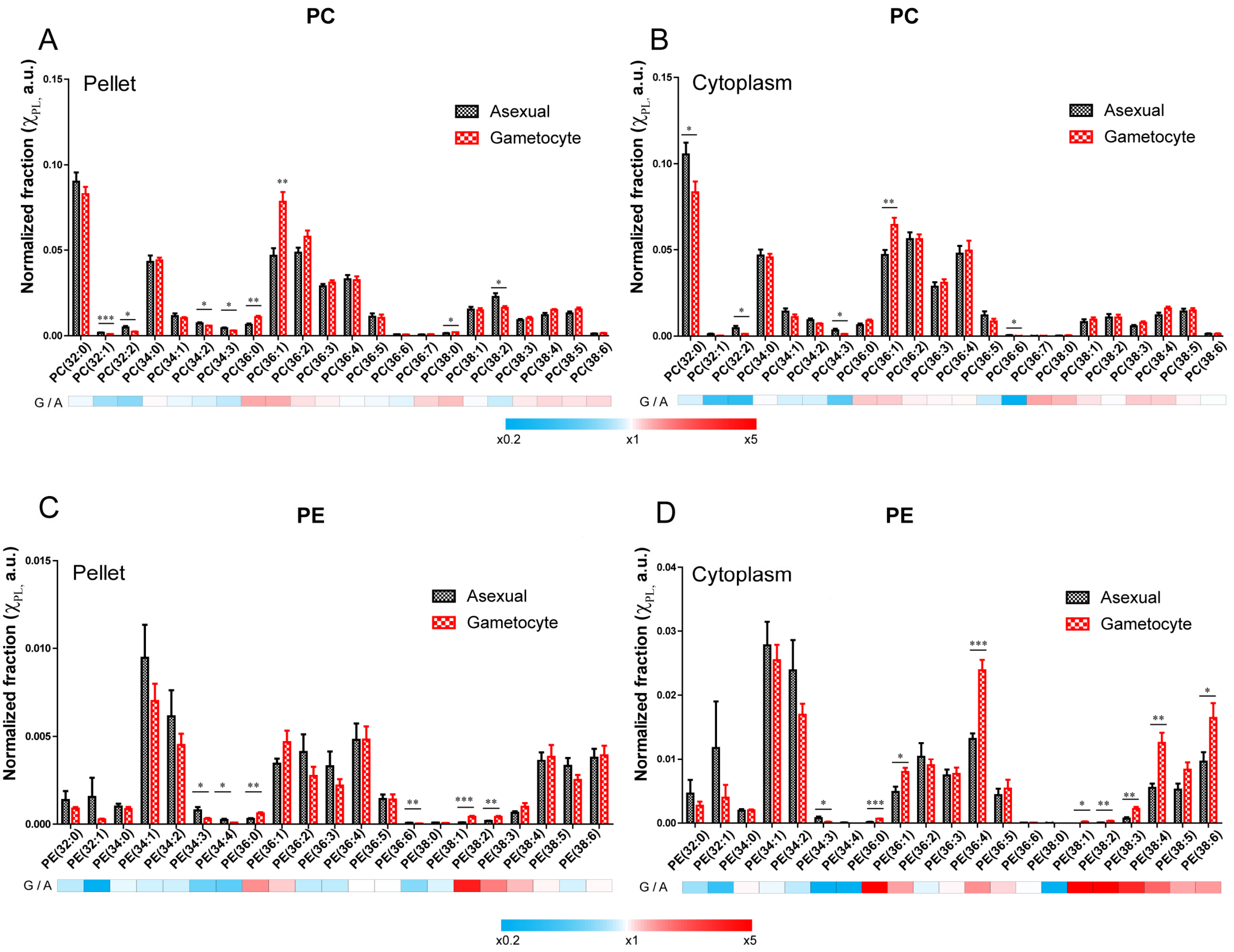

Figure S1. Phospholipid profiles of asexual and gametocyte stages grown in human serum. (A, B) Normalized fractions (χ_{PL}) of 22 PC from the pellet and cytoplasmic samples. (C, D) Normalized fractions of 21 PE from the pellet and cytoplasmic samples. Fraction values were calculated using total phospholipids in the asexual and gametocyte sample sets (n=6). For the color bar representation of fractional amounts below each panel, gametocyte (G) and asexual stage (A) ratios were assigned to an 8-bit color scale from blue (G/A <1, white: G/A=1) to red (G/A >1) and mapped to 40 red and 40 blue color steps from G/A = 5 to 0.2, respectively (step size = 6.4).

In results from the PC species, pellet samples showed higher fractional values of PC(32:1), PC(32:2), PC(34:2), PC(34:3), and PC(38:2) in asexual stages, whereas PC(36:0), PC(36:1), and PC(38:0) were greater in gametocytes (Supplemental Table S2). From the cytoplasmic samples, higher levels of saturated PC(32:0) were detected in the asexual stages, whereas unsaturated PC(36:1) was greater in gametocyte stages; small fractions of PC(32:2), PC(34:3), and PC(36:6) also showed somewhat higher levels in asexual stages (Supplemental Table S2). In results from the PE species, relatively low levels of PE(34:3), PE(34:4), and PE(36:6) were statistically higher in asexual stages, while PE(36:0), PE(38:1), and PE(38:2) were higher in the gametocyte stages (Supplemental Table S3). Higher levels of PE(36:0), PE(36:1), PE(36:4), PE(38:1), PE(38:3), PE(38:4), and PE(38:6) were more abundant in the cytoplasm of gametocytes; only a low level of PE(34:3) showed statistically significant greater signal from the cytoplasm of asexual stages (Supplemental Table S3). *p<0.05, **p<0.01, ***p<0.001 by two-tailed t-test.

Biology Open • Supplementary information

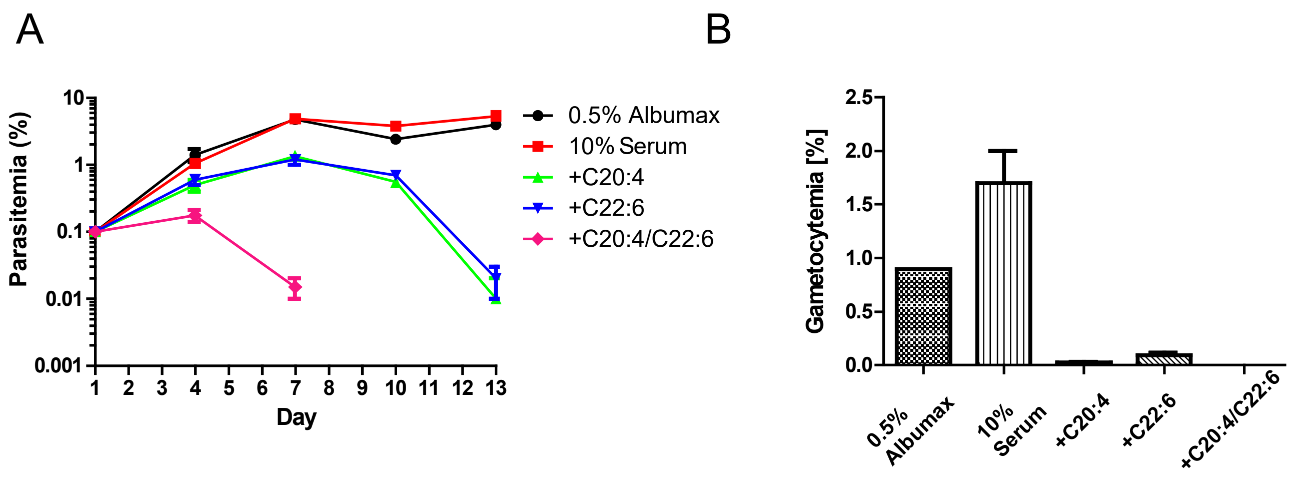

Figure S2. **Asexual stage propagation and gametocytogenesis adversely affected by the addition of FFA in EtOH/$CHCl_3$ solvent to culture.** Continuous addition of FFA (13 µg/mL of C20:4, and 8 µg/mL of C22:6) in EtOH/$CHCl_3$ resulted in severe reductions of both asexual parasitemia (left) and gametocytemia (right) in AlbuMAX-supported cultures.

Biology Open • Supplementary information

Biology Open (2019): doi:10.1242/bio.042259: Supplementary information

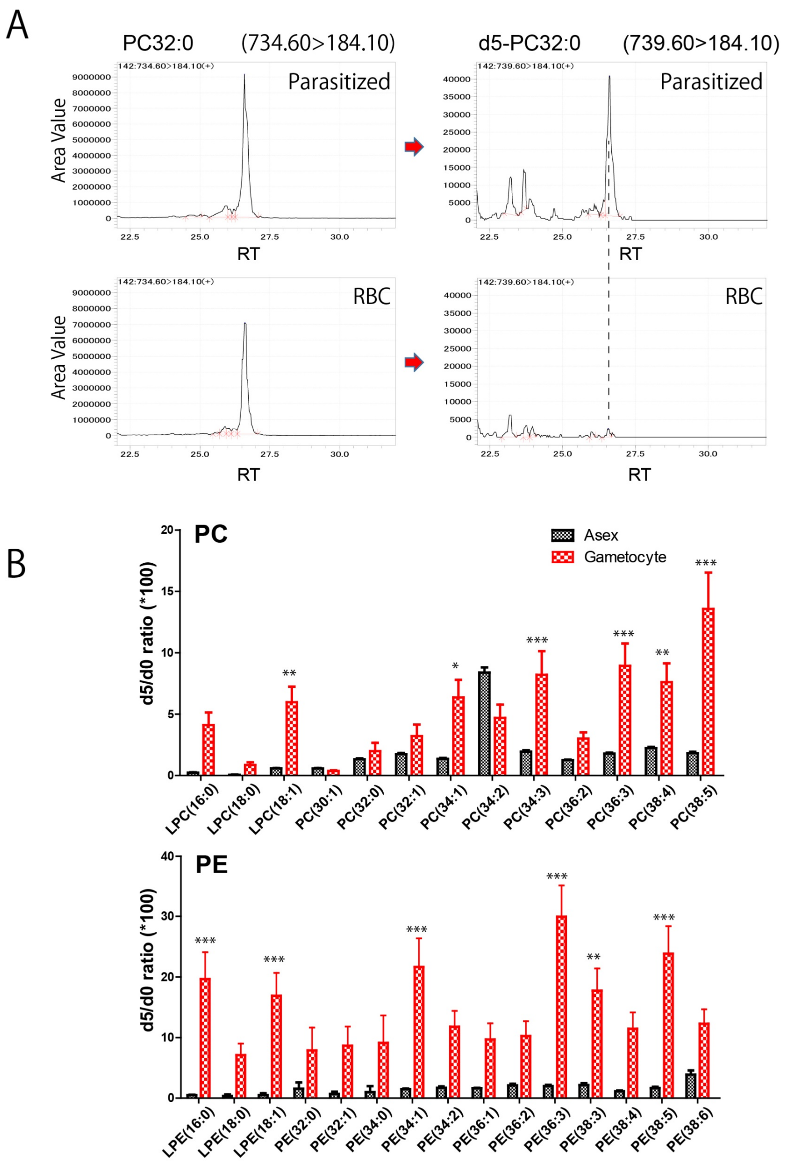

Figure S3. Fatty acid incorporation analysis by deuterated glycerol labeling. 5 mM deuterium-labeled glycerol (Glycerol-1,1,2,3,3,-d5, 98% isotopic purity; Sigma) with 1mM glucose was added to the glucose-free culture media and allowed parasites to convert glycerol into glycerol-3-phosphate (G3P) by glycerol kinase and combine fatty acids into phospholipids. Unlabeled glycerol was added to control cultures. The lipid extracts were obtained after 24 hr and hr and labeled phospholipids were analyzed using LC-MS. Sample concentrations were adjusted to avoid signal saturation based on test analyses of same samples that were performed prior to the all data collection runs. (A) An example of chromatograms that demonstrates fatty acid incorporation into glycerol by parasites. Left panels: detection of intrinsic PC32:0 but no d5 signal. Right panels: detection of d5-labeled PC32:0 showing that fatty acids have been incorporated into d5-glycerol. The negligible d5 signal from non-parasitized erythrocytes confirms that erythrocytes do not convert glycerol into phospholipid. (B) Fractional lipid amounts of PC and PE species in the asexual stages and gametocyte. The d5/d0 (d5-labeled/non-labeled phospholipid, ×100, a.u.) ratios provide a visual comparison of relative amounts of each species in asexual and gametocyte stages. Ratios are shown for 13 PCs including lysophospholipids (LPL) and 15 PEs that were recognized in d5 channels. PC and PE species showed overall greater uptake in gametocytes than in asexual stages. Among the PC species, relative glycerol incorporation was significantly higher with the unsaturated lipids, lyso PC(18:1) ($d5/d0_{asex}$ *vs.* $d5/d0_{game}$ = 0.58 *vs.* 5.97), PC(34:1) (1.37 *vs.* 6.37), PC(34:3) (1.95 *vs.* 8.21), PC(36:3) (1.78 *vs.* 8.94), PC(38:4) (2.25 *vs.* 7.61), and PC(38:5) (1.83 *vs.* 13.57). Among PE species, significantly higher gametocyte uptake was observed with two lyso PEs, LPE(16:0) (0.48 *vs.* 19.64) and LPE(18:1) (0.48 *vs.* 16.88), and with PE(34:1) (1.48 *vs.* 21.65), PE(36:3) (2.00 *vs.* 29.93), PE(38:3) (2.15 *vs.* 17.74) and PE(38:5) (1.66 *vs.* 23.83). *p<0.05, **p<0.01, ***p<0.001 by two-way ANOVA with Bonferroni test.

Biology Open • Supplementary information

			Mean				Significance		CI of difference		n
			Asexual	std. err.	Gametocyte	std err.	t	*p*	Lower	Upper	
Pellet	MAG	16:0	0.106	0.025	0.027	0.009	2.960	0.0141	0.020	0.139	6
		18:0	0.168	0.040	0.030	0.013	3.269	0.0084	0.044	0.232	6
	DAG	32:0	0.023	0.003	0.008	0.001	5.541	0.0002	0.009	0.021	6
		34:0	0.016	0.004	0.003	0.001	3.246	0.0088	0.004	0.022	6
		34:1	0.115	0.019	0.205	0.032	2.417	0.0362	-0.173	-0.007	6
		34:2	0.104	0.015	0.120	0.011	0.841	0.4201	-0.056	0.025	6
		36:0	0.021	0.008	0.002	0.001	2.377	0.0388	0.001	0.038	6
		36:1	0.061	0.011	0.126	0.006	5.177	0.0004	-0.093	-0.037	6
		36:2	0.061	0.009	0.079	0.008	1.527	0.1578	-0.045	0.008	6
		36:4	0.074	0.013	0.108	0.006	2.405	0.0370	-0.064	-0.002	6
		38:4	0.108	0.016	0.183	0.022	2.773	0.0197	-0.134	-0.015	6
Cytoplasm	MAG	16:0	0.190	0.014	0.213	0.027	0.736	0.4790	-0.091	0.047	6
		18:0	0.490	0.044	0.468	0.029	0.408	0.6917	-0.096	0.014	6
	DAG	32:0	0.019	0.005	0.010	0.002	1.598	0.1410	-0.004	0.022	6
		34:0	0.049	0.013	0.043	0.008	0.364	0.7232	-0.029	0.040	6
		34:1	0.016	0.005	0.013	0.002	0.565	0.5846	-0.010	0.017	6
		34:2	0.012	0.007	0.009	0.003	0.366	0.7223	-0.014	0.020	6
		36:0	0.122	0.018	0.122	0.014	0.004	0.9973	-0.050	0.050	6
		36:1	0.017	0.008	0.010	0.004	0.736	0.4630	-0.013	0.027	6
		36:2	0.007	0.004	0.008	0.003	0.080	0.9377	-0.011	0.011	6
		36:4	0.007	0.003	0.013	0.007	0.780	0.4524	-0.023	0.001	6
		38:4	0.006	0.003	0.025	0.015	1.208	0.2547	-0.052	0.015	6

Table S1. Summary of MAG and DAG fractional values.

Biology Open • Supplementary information

PC		Mean				Significance		CI of difference		n
		Asexual	std. err.	Gametocyte	std err.	t	*p*	Lower	Upper	
Pellet	PC(32:0)	0.09023	0.00537	0.08291	0.00420	1.07500	0.30780	-0.00786	0.02251	6
	PC(32:1)	0.00179	0.00013	0.00097	0.00004	6.09400	0.00010	0.00050	0.00112	6
	PC(32:2)	0.00485	0.00083	0.00230	0.00019	3.00400	0.01320	0.00066	0.00444	6
	PC(34:0)	0.04315	0.00369	0.04399	0.00159	0.20860	0.83890	-0.00979	0.00811	6
	PC(34:1)	0.01148	0.00160	0.01028	0.00061	0.70320	0.49800	-0.00262	0.00503	6
	PC(34:2)	0.00726	0.00061	0.00577	0.00023	2.26800	0.04670	0.00003	0.00295	6
	PC(34:3)	0.00436	0.00048	0.00294	0.00019	2.75300	0.02040	0.00027	0.00256	6
	PC(36:0)	0.00639	0.00081	0.01089	0.00071	4.18000	0.00190	-0.00690	-0.00210	6
	PC(36:1)	0.04672	0.00438	0.07817	0.00601	4.22800	0.00170	-0.04802	-0.01487	6
	PC(36:2)	0.04851	0.00292	0.05783	0.00357	2.02300	0.07060	-0.01959	0.00094	6
	PC(36:3)	0.02885	0.00137	0.03108	0.00130	1.18400	0.26400	-0.00643	0.00197	6
	PC(36:4)	0.03299	0.00245	0.03245	0.00230	0.16160	0.87480	-0.00695	0.00803	6
	PC(36:5)	0.01111	0.00197	0.01048	0.00189	0.22930	0.82330	-0.00545	0.00670	6
	PC(36:6)	0.00078	0.00012	0.00066	0.00010	0.76980	0.45920	-0.00022	0.00046	6
	PC(36:7)	0.00065	0.00013	0.00085	0.00010	1.18500	0.26330	-0.00056	0.00017	6
	PC(38:0)	0.00138	0.00024	0.00203	0.00007	2.62200	0.02550	-0.00121	-0.00010	6
	PC(38:1)	0.01525	0.00162	0.01486	0.00129	0.18790	0.85470	-0.00422	0.00500	6
	PC(38:2)	0.02271	0.00211	0.01627	0.00102	2.74300	0.02070	0.00121	0.01166	6
	PC(38:3)	0.00909	0.00073	0.01020	0.00085	1.00100	0.34030	-0.00360	0.00137	6
	PC(38:4)	0.01201	0.00135	0.01505	0.00057	2.08100	0.06410	-0.00631	0.00021	6
	PC(38:5)	0.01307	0.00107	0.01535	0.00111	1.48000	0.16980	-0.00570	0.00115	6
	PC(38:6)	0.00119	0.00027	0.00149	0.00022	0.86040	0.40970	-0.00108	0.00048	6
Cytoplasm	PC(32:0)	0.08350	0.00620	0.02192	0.00924	2.37200	0.03910	0.00133	0.04252	6
	PC(32:1)	0.00109	0.00042	0.00031	0.00008	1.81600	0.09940	-0.00018	0.00174	6
	PC(32:2)	0.00458	0.00137	0.00121	0.00007	2.45800	0.03380	0.00031	0.00642	6
	PC(34:0)	0.04667	0.00341	0.04555	0.00209	0.28040	0.78490	-0.00778	0.01002	6
	PC(34:1)	0.01418	0.00187	0.01097	0.00165	1.28800	0.22680	-0.00234	0.00875	6
	PC(34:2)	0.00925	0.00094	0.00716	0.00031	2.11700	0.06030	-0.00011	0.00429	6
	PC(34:3)	0.00333	0.00095	0.00116	0.00016	2.25400	0.04780	0.00003	0.00432	6
	PC(36:0)	0.00639	0.00092	0.00893	0.00068	2.22100	0.05060	-0.00509	0.00001	6
	PC(36:1)	0.04696	0.00289	0.06432	0.00418	3.41500	0.00660	-0.02869	-0.00604	6
	PC(36:2)	0.05616	0.00390	0.05616	0.00278	0.00136	0.99890	-0.01067	0.01068	6
	PC(36:3)	0.02862	0.00258	0.03090	0.00202	0.69610	0.50220	-0.00958	0.00502	6
	PC(36:4)	0.04767	0.00457	0.04950	0.00574	0.24950	0.80800	-0.01822	0.01455	6
	PC(36:5)	0.01198	0.00233	0.00851	0.00158	1.23800	0.24420	-0.00278	0.00974	6
	PC(36:6)	0.00047	0.00016	0.00009	0.00004	2.27800	0.04590	0.00001	0.00077	6
	PC(36:7)	0.00013	0.00006	0.00022	0.00003	1.37900	0.19870	-0.00025	0.00006	6
	PC(38:0)	0.00027	0.00012	0.00043	0.00013	0.88320	0.39790	-0.00054	0.00023	6
	PC(38:1)	0.00811	0.00163	0.00949	0.00137	0.64780	0.53170	-0.00613	0.00337	6
	PC(38:2)	0.01077	0.00205	0.01081	0.00156	0.01676	0.98700	-0.00578	0.00570	6
	PC(38:3)	0.05682	0.00077	0.00777	0.00083	1.85200	0.09380	-0.00460	0.00042	6
	PC(38:4)	0.01203	0.00160	0.01600	0.00109	2.05200	0.06730	-0.00827	0.00034	6
	PC(38:5)	0.01427	0.00158	0.01488	0.00136	0.29280	0.77560	-0.00525	0.00403	6
	PC(38:6)	0.00127	0.00035	0.00123	0.00044	0.07511	0.94160	-0.00120	0.00128	6

Table S2. Fractional area values of phosphatidylcholine species.

Biology Open • Supplementary information

PE		Mean				Significance		CI of difference		n
		Asexual	std. err.	Gametocyte	std err.	t	*p*	Lower	Upper	
Pellet	PE(32:0)	0.00137	0.00051	0.00088	0.00009	0.93320	0.37270	-0.00067	0.00164	6
	PE(32:1)	0.00154	0.00109	0.00027	0.00004	1.16000	0.27300	-0.00117	0.00370	6
	PE(34:0)	0.00100	0.00016	0.00085	0.00013	0.69840	0.50080	-0.00032	0.00061	6
	PE(34:1)	0.00100	0.00016	0.00015	0.00021	0.69840	0.50080	-0.00032	0.00061	6
	PE(34:2)	0.00946	0.00187	0.00702	0.00097	1.15900	0.27330	-0.00226	0.00715	6
	PE(34:3)	0.00614	0.00148	0.00452	0.00064	1.01000	0.33640	-0.00197	0.00522	6
	PE(34:4)	0.00079	0.00019	0.00031	0.00005	2.50000	0.03150	0.00005	0.00091	6
	PE(36:0)	0.00030	0.00004	0.00060	0.00007	3.95600	0.00270	-0.00048	-0.00013	6
	PE(36:1)	0.00343	0.00031	0.00468	0.00065	1.72200	0.11570	-0.00125	-0.00286	6
	PE(36:2)	0.00412	0.00100	0.00273	0.00053	1.22700	0.24790	-0.00113	0.00391	6
	PE(36:3)	0.00329	0.00086	0.00219	0.00036	1.18100	0.26500	-0.00097	0.00317	6
	PE(36:4)	0.00481	0.00093	0.00483	0.00075	0.01121	0.99130	-0.00266	0.00264	6
	PE(36:5)	0.00143	0.00025	0.00140	0.00029	0.07000	0.94560	-0.00083	0.00088	6
	PE(36:6)	0.00007	0.00001	0.00003	0.00001	2.47400	0..0329	0.00000	0.00007	6
	PE(38:0)	0.00008	0.00001	0.00007	0.00001	1.20400	0.25630	-0.00001	0.00001	6
	PE(38:1)	0.00010	0.00001	0.00041	0.00006	5.12700	0.00040	-0.00045	-0.00018	6
	PE(38:2)	0.00018	0.00002	0.00040	0.00006	3.46200	0.00610	-0.00035	-0.00080	6
	PE(38:3)	0.00065	0.00089	0.00099	0.00021	1.50200	0.16400	-0.00085	0.00016	6
	PE(38:4)	0.00361	0.00049	0.00384	0.00068	0.26940	0.79310	-0.00209	-0.00164	6
	PE(38:5)	0.00331	0.00047	0.00251	0.00028	1.44900	0.17800	-0.00043	0.00202	6
	PE(38:6)	0.00380	0.00051	0.00394	0.00054	0.19230	0.85130	-0.00178	0.00150	6
Cytoplasm	PE(32:0)	0.00461	0.00213	0.00273	0.00062	0.84600	0.41730	-0.00307	0..006823	6
	PE(32:1)	0.01178	0.00725	0.00397	0.00199	1.03900	0.32320	-0.00894	0.02456	6
	PE(34:0)	0.00194	0.00022	0.00198	0.00015	0.17550	0.86420	-0.00064	0.00055	6
	PE(34:1)	0.02748	0.00366	0.02547	0.00238	0.52620	0.61020	-0.00744	0.01204	6
	PE(34:2)	0.02388	0.00470	0.01660	0.00171	1.38400	0.19640	-0.00422	0.01805	6
	PE(34:3)	0.00078	0.00024	0.00017	0.00007	0.00061	0.00025	0.00005	0.00117	6
	PE(34:4)	0.00007	0.00004	n.d.	n.d.	---	---	---	---	6
	PE(36:0)	0.00017	0.00005	0.00067	0.00005	7.15500	<0.0001	-0.00065	-0.00037	6
	PE(36:1)	0.00486	0.00082	0.00797	0.00063	3.00200	0.01330	-0.00543	0.00080	6
	PE(36:2)	0.01040	0.00213	0.00903	0.00094	0.58640	0.57060	-0.00382	0.00655	6
	PE(36:3)	0.00742	0.00094	0.00768	0.00098	0.18810	0.85450	-0.00328	0.00277	6
	PE(36:4)	0.01318	0.00087	0.02390	0.00159	5.90100	0.00020	-0.01476	-0.00667	6
	PE(36:5)	0.00436	0.00101	0.00538	0.00137	0.60260	0.56020	-0.00482	0.00277	6
	PE(36:6)	0.00005	0.00003	0.00005	0.00002	0.01075	0.99160	-0.00009	0.00009	6
	PE(38:0)	0.00002	0.00001	0.00000	0.00000	1.19400	0.26020	-0.00001	0.00004	6
	PE(38:1)	0.00000	0.00000	0.00021	0.00008	2.67500	0.02330	-0.00037	0.00003	6
	PE(38:2)	0.00007	0.00026	0.00032	0.00007	3.29300	0.00810	-0.00041	0.00008	6
	PE(38:3)	0.00070	0.00023	0.00223	0.00030	4.05800	0.00230	-0.00237	0.00069	6
	PE(38:4)	0.00550	0.00065	0.01257	0.00158	4.13700	0.00200	-0.01087	0.00326	6
	PE(38:5)	0.00521	0.00096	0.00833	0.00112	2.21000	0.06000	-0.00640	0.00016	6
	PE(38:6)	0.00959	0.00152	0.01646	0.00230	2.48900	0.03200	-0.01302	0.00072	6

Table S3. Fractional area values of phosphatidylethanolamines
